# Lipidomics reveals that sustained SREBP-1-dependent lipogenesis is a key mediator of gefitinib-acquired resistance in EGFR-mutant lung cancer

**DOI:** 10.1038/s41420-021-00744-1

**Published:** 2021-11-13

**Authors:** Chuncao Xu, Lei Zhang, Daifei Wang, Shiqin Jiang, Di Cao, Zhongxiang Zhao, Min Huang, Jing Jin

**Affiliations:** 1 Guangdong Provincial Key Laboratory of New Drug Design and Evaluation, School of Pharmaceutical Sciences, Sun Yat-sen University, Guangzhou, 510006 China; 2 School of Chinese Materia Medica, Guangzhou University of Chinese Medicine, Guangzhou, 510006 China

**Keywords:** Cancer metabolism, Non-small-cell lung cancer

## Abstract

Patients with EGFR mutations in non-small cell lung cancer (NSCLC) have been greatly benefited from gefitinib, however, the therapeutic has failed due to the presence of acquired resistance. In this study, we show that gefitinib significantly induces downregulation of Sterol Regulator Element Binding (SREBP1) in therapy-sensitive cells. However, this was not observed in EGFR mutant NSCLC cells with acquired resistance. Lipidomics analysis showed that gefitinib could differently change the proportion of saturated phospholipids and unsaturated phospholipids in gefitinib-sensitive and acquired-resistant cells. Besides, levels of ROS and MDA were increased upon SREBP1 inhibition and even more upon gefitinib treatment. Importantly, inhibition of SREBP1 sensitizes EGFR-mutant therapy-resistant NSCLC to gefitinib both in vitro and in vivo models. These data suggest that sustained de novo lipogenesis through the maintenance of active SRBEP-1 is a key feature of acquired resistance to gefitinib in EGFR mutant lung cancer. Taken together, targeting SREBP1-induced lipogenesis is a promising approach to overcome acquired resistance to gefitinib in EGFR-mutant lung cancer.

## Introduction

Lung cancer is the most common malignant tumor in the world and non-small cell lung cancer (NSCLC) accounts for 85% [1]. Recently, the molecular targeted therapy has become an important treatment method in the treatment of EGFR mutant NSCLC [2]. Gefitinib as the representative of the first generation of EGFR-TKI has achieved positive clinical efficacy [3]. However, drug resistance which most patients will develop after 10–14 months of administration badly limits its continual application [4]. Although the new generation of EGFR-TKIs has been developed, gefitinib is still widely used due to its lower price and being recommended as a first-line agent in patients with newly diagnosed stage IV NSCLC with EGFR-positive mutations by the Chinese Society of Clinical Oncology Guidelines (CSCO) 2020. Many mechanisms have been described in gefitinib-induced resistance [5–8]. Different mutational events can be selected in different drug-resistant clones from the same patient and even co-occur within the same pathological changes [9]. Therefore, it is necessary to improve the effectiveness of gefitinib, for example, co-targeting with other essential cancer pathways or key mediators of EGFR signaling itself.

One of the signaling pathways that play an essential role in multiple oncogenic processes is de novo lipogenesis [10]. In order to satisfy the rapid proliferation of tumor cells, the lipid synthesis is enhanced in many cancers [11]. It is found that lipid metabolism is very active in many tumor cells with drug resistance to targeted therapy [10, 12]. In tumor cells, the main source of lipid is the de novo synthesis. SREBP1 is an important transcription factor in the de novo synthesis of lipids [13]. It can activate fatty acid synthase (FASN), insulin-induced gene 1 (INSIG1), acyl CoA carboxylase (ACC), and stearoyl COA desaturase (SCD) [14]. It regulates the biosynthesis of phospholipids, fatty acids, and triglycerides. Abnormal activation of the lipogenetic pathway in cancer is necessary for the synthesis of phospholipids, which is an important component of cell membranes and that supports cell growth and proliferation [15, 16]. SREBP1-mediated lipid synthesis pathway mainly produces saturated fatty acids and monounsaturated fatty acids. In many tumor cells, the proportion of saturated and monounsaturated fatty acids on the cell membrane increases, which makes tumor cells less susceptible to lipid peroxidation, thereby providing a survival advantage to cancer cells that were exposed to anticancer drugs and oxidative stress damage [17]. It has been reported that inhibition of SREBP-1 increases gefitinib sensitivity in lung cancer A549 and PC9 cells [18, 19]. However, the role of SREBP1 mediated lipogenesis in gefitinib-acquired resistance has not elucidated yet.

In the current study, we show that SREBP-1 mediated lipogenic pathway is a key mediator of oncogenic EGFR and that its constitutive activation contributes to gefitinib-acquired resistance in EGFR mutant lung cancer. Our findings support that the combination use of SREBP1 inhibitors is a novel strategy to overcome gefitinib-acquired resistance.

## Results

### De novo lipogenesis is activated in EGFR mutant NSCLC cells with gefitinib-acquired resistance

**Fig. 1:**
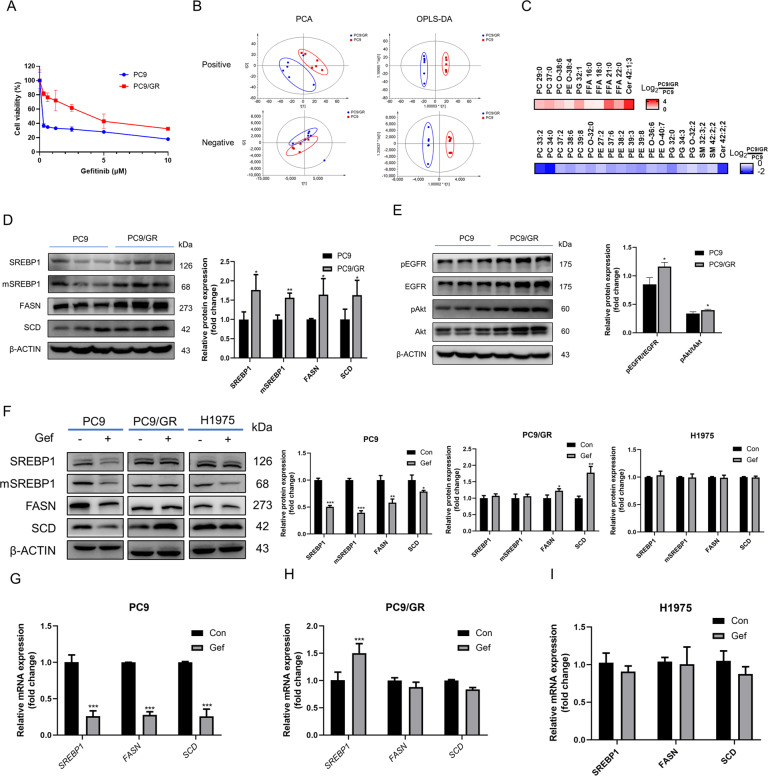
Effects of gefitinib on lipid metabolism involve SREBP1. **A** PC9 and PC9/GR cells were treated with gefitinib for 72 h, and the cell viability was measured by CCK8. **B** Lipidomic analysis on PC9 and PC9/GR cells in positive and negative ion modes, respectively. PCA and OPLS-DA score plots of lipidomic profiles obtained from LC-MS/MS. **C** Comparison of the changed lipids between PC9/GR and PC9 group. Heatmap shows the ratios of phospholipid species abundance in cells. Species are represented by the total number of fatty acid carbons and unsaturated carbons, which are linked by a colon. **D** The protein expression level of SREBP1 and relevant protein were assessed in PC9 and PC9/GR cells. **E** The protein expression level of EGFR and relevant protein were assessed in PC9 and PC9/GR cells. **F** Western Blot analysis of PC9, PC9/GR, and H1975 cells treated with gefitinib. **G**–**I** The mRNA levels of *SREBP1, FASN*, and *SCD* were determined by RT-qPCR. Data are expressed as the mean ± SD (*n* = 3). *P < 0.05, **P < 0.01, ***P < 0.001 compared with control.

It has been shown that de novo lipogenesis is activated in lung cancer [20]. To identify the resistance-associated lipid pathway signatures, we compared PC9 cells and PC9/GR cells by lipidomics. Principal component analysis of PC9 and PC9/GR cells showed a clear difference (Fig. 1B). OPLS-DA was used to further investigate the supervised data analysis for identifying differential metabolites (Fig. 1C). It is notable that many lipid species were altered in PC9/GR cells according to normalized heatmap pictures. As SREBP1 is an important transcription factor in the de novo synthesis of lipids, we found that the protein levels of SREBP1 and its downstream targets, FASN and SCD in PC9/GR were upregulated 76%, 57%, 64 and 63%, respectively, when compared to PC9 cells (Fig. 1D). What’s more, the protein levels of pEGFR/EGFR and pAkt/Akt were upregulated in PC9/GR cells (Fig. 1E).

To investigate whether a link exists between constitutively active SREBP1 signaling and growth of cells with gefitinib resistance, PC9, PC9/GR and H1975 cells were exposed to gefitinib for 72 h. From hereon, the gefitinib doses used for PC9 cells were 0.1 μM and 1 μM for both PC9/GR and H1975 cells, respectively. The expression of SREBP1 (mature form), FASN, and SCD in PC9 cells with gefitinib treatment were decreased 74.1%, 72.2%, and 74.4%, respectively, while this was not observed in the therapy-resistant lines (Fig. 1F). The mRNA levels of *SREBP1, FASN*, and *SCD* were decreased upon gefitinib exposure in PC9 (49.4, 60.4, 41.6, and 21.3%), but not in resistant cell lines (Fig. 1G–I). Taken together, gefitinib inhibits the activation of SREBP-1 in PC9, but not in PC9/GR or H1975 cells, which indicates that lipogenesis is sustained in therapy-resistant cells when compared to therapy-sensitive cells upon gefitinib treatment.

**Fig. 2:**
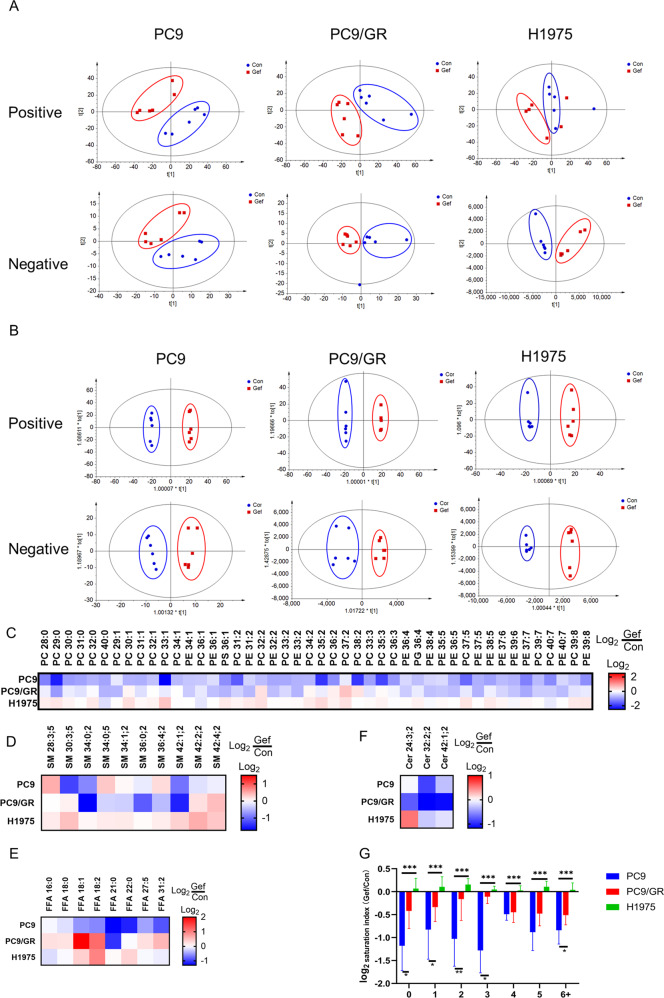
De novo lipogenesis in gefitinib-sensitive PC9 cells is inhibited by gefitinib, while remains activate in PC9/GR and H1975 cells. **A** Lipidomic analysis on control and Gef group in positive and negative ion modes, respectively. PCA score plots of lipidomic profiles obtained from LC-MS/MS. **B** OPLS-DA score plots of lipidomic profiles obtained from LC-MS/MS. **C**–**F** Comparison of the changed lipids between control and Gef group. Heatmap shows the ratios of phospholipid species abundance in cells treated with and without gefitinib. Species are represented by the total number of fatty acid carbons and unsaturated carbons, which are linked by a colon. **G** Log_2_ ratios of the changes in saturation index induced by gefitinib. The saturation index is derived from the sum of species with the same unsaturated level. Data are expressed as the mean ± SD (*n* = 6).

PCA and OPLS-DA of PC9, PC9/GR, and H1975 cells showed a clear difference upon gefitinib treatment (Fig. 2A, B). Many lipid species were altered in gefitinib treated group according to normalized heatmap pictures. PC/PE/PS/PG were the most abundant lipid species that were altered significantly and the content of most of them were downregulated in PC9 cells. However, this was not observed in PC9/GR cells and their content were even upregulated in H1975 cells (Fig. 2C). SMs were significantly altered with a different variation trend in PC9 cells. SMs were reduced apparently in PC9/GR cells, but they were increased in H1975 cells (Fig. 2D). FAs were reduced in PC9 cells, but such change was not observed in PC9/GR cells and H1975 cells (Fig. 2E). Cers were both reduced in PC9 and PC9/GR cells, but not in H1975 cells (Fig. 2F). After gefitinib treatment, saturated and monounsaturated phospholipids were decreased more significantly in PC9 cells, but not in H1975 and PC9/GR cells. For the comparison of polyunsaturated phospholipids, the decrease ratio of phospholipids with high saturation in PC9 cells was lower than that of phospholipids with low saturation, while it was opposite in PC9/GR cells (Fig. 2G). Taken together, these results suggest that gefitinib inhibits de novo lipogenesis in gefitinib-sensitive cells but not in resistant cells.

### Inhibition of SREBP-1 sensitizes EGFR-TKI resistant cells to gefitinib

**Fig. 3:**
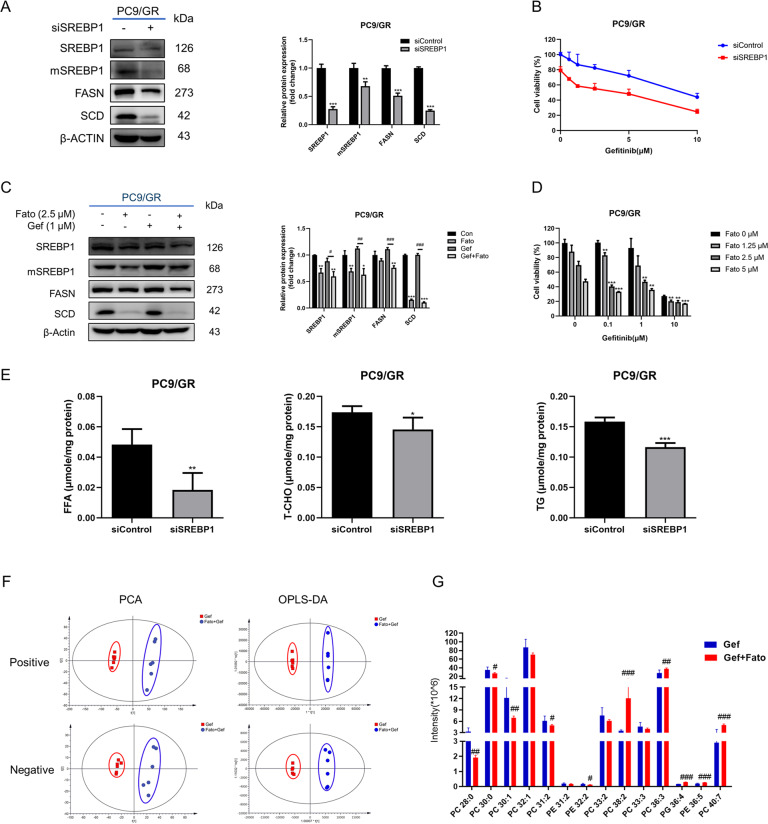
Inhibition of SREBP1 makes gefitinib-acquired resistant cells PC9/GR more sensitive to gefitinib. **A** Western blot analysis for SREBP1, mSREBP1, FASN and SCD in PC9/GR cells transfected with si-SREBP1 (*n* = 3). **B** PC9/GR cells transfected with si-control or si-SREBP1 were cultured for 72 h with various concentrations of gefitinib, and the cell viability was determined by CCK-8 assay (*n* = 5). **C** Western blot analysis for SREBP1, mSREBP1, FASN, and SCD in PC9/GR cells treated with gefitinib in the absence or presence of Fato (*n* = 3). **D** PC9/GR cells were treated with gefitinib alone or in combination with different concentrations of Fato for 72 h, and cell viability was determined (*n* = 5). **E** Cell lipids were extracted, and TG, T-CHO and FFA were measured (*n* = 4). Data are expressed as the mean ± SD. **F** Lipidomic analysis on Gef and Gef + Fato group in positive and negative ion modes, respectively. PCA and OPLS-DA score plots of lipidomic profiles obtained from LC-MS/MS. **G** Comparison of the changed lipids between Gef and Gef + Fato group. *P < 0.05, **P < 0.01, ***P < 0.001 compared with control. ^*#*^*P* < 0.05, ^*##*^*P* < 0.01, ^*###*^*P* < 0.001 compared with gefitinib.

To investigate the effects of SREBP1 and its downstream genes on the sensitivity of EGFR-TKI resistant cells to gefitinib, we evaluated the effect of SREBP1 knockdown on FASN and SCD expression in PC9 and PC9/GR cells. SREBP1 silencing was shown to strongly reduce FASN (48.9%) and SCD (75.2%) levels in PC9/GR cells as compared to untreated cells (Fig. 3A). As expected, silencing SREBP1 also reduced the cell growth of PC9/GR upon exposure to gefitinib (Fig. 3B). To confirm whether inhibition of SREBP1 by blocking proteolytic cleavage has similar effects as silencing SREBP1 directly. Consistent with previous reports [21], treating cells with fatostatin markedly decreased the expression levels of mature form of SREBP1 in PC9/GR cells (31.8%). Similar as those observed in SREBP1 knockdown cells, fatostatin-induced inhibition of SREBP1 activation significantly reduced the expression of downstream target genes which are important for lipid biogenesis (Fig. 3C). Proliferation of PC9/GR cells was strongly inhibited with co-treatment of gefitinib and fatostatin (Fig. 3D).

In order to verify the effect of silencing SREBP1 on the major lipid components in gefitinib-resistant cells, we measured the intracellular levels of FFA, T-CHO, and TG (Fig. 3E). They were significantly decreased in SREBP1 knockdown cells, which were consistent with the results of protein expression. PCA and OPLS-DA of PC9/GR cells showed a clear difference between gef and gef + fato treatment (Fig. 3F). Chemical inhibition of SREBP1 depleted mono-unsaturated and fully saturation phospholipid species and increased membrane poly-unsaturation (Fig. 3G). These data indicate that SREBP1 inhibition sensitizes therapy-resistant NSCLC cells to gefitinib therapy.

### Downregulation of SREBP1-induced lipid peroxidation in gefitinib-resistant cells

**Fig. 4:**
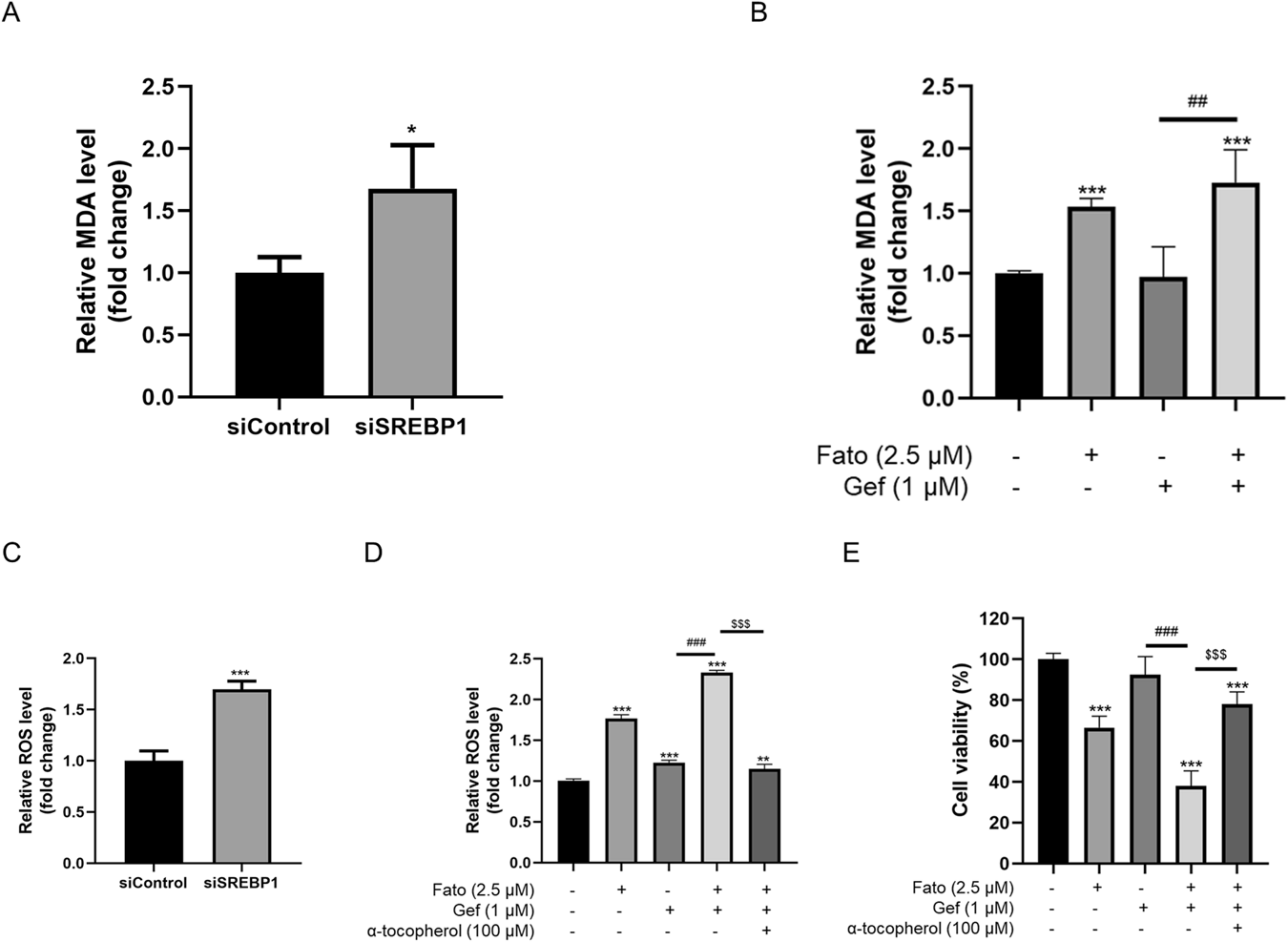
Inhibition of SREBP1 affects lipid peroxidation and oxidative stress in PC9/GR cells. PC9/GR cells transfected with siRNA or were treated with gefitinib in the presence or absence of Fato (2.5 μM) and α-tocopherol (100 μM) for 72 h. **A**, **B** Intracellular MDA content measurement of PC9/GR cells. **C**, **D** ROS concentration of PC9/GR cells were measured. **E** Cell viability was measured by CCK8. Data are expressed as the mean ± SD (*n* = 4). *P < 0.05, **P < 0.01, ***P < 0.001 compared with control. ^*#*^*P* < 0.05, ^*##*^*P* < 0.01, ^*###*^*P* < 0.001 compared with gefitinib. ^*$$$*^*P* < 0.001 compared with Gef + Fato.

To better understand the mechanism underlying the relationship between SREBP1 and lipid peroxidation [12], we measured the levels of ROS and MDA. Levels of MDA increased upon exposure to fatostatin or silencing SREBP1. Co-treatment with fatostatin and gefitinib resulted in a further increase in MDA levels (Fig. 4A, B). MDA is a direct by-product of lipid peroxidation. ROS has been linked to membrane lipid peroxidation, which results in the accumulation of toxic by-products [22]. The levels of ROS were significantly increased upon inhibition of SREBP1 (Fig. 4C, D). Under combined fatostatin and gefitinib, the addition of antioxidant partially rescued cell viability (Fig. 4E). These findings indicate that SREBP1 inhibition sensitizes cells to gefitinib partly through alterations of lipid peroxidation.

### SREBP-1 inhibition sensitizes carcinoma to gefitinib in vivo

**Fig. 5:**
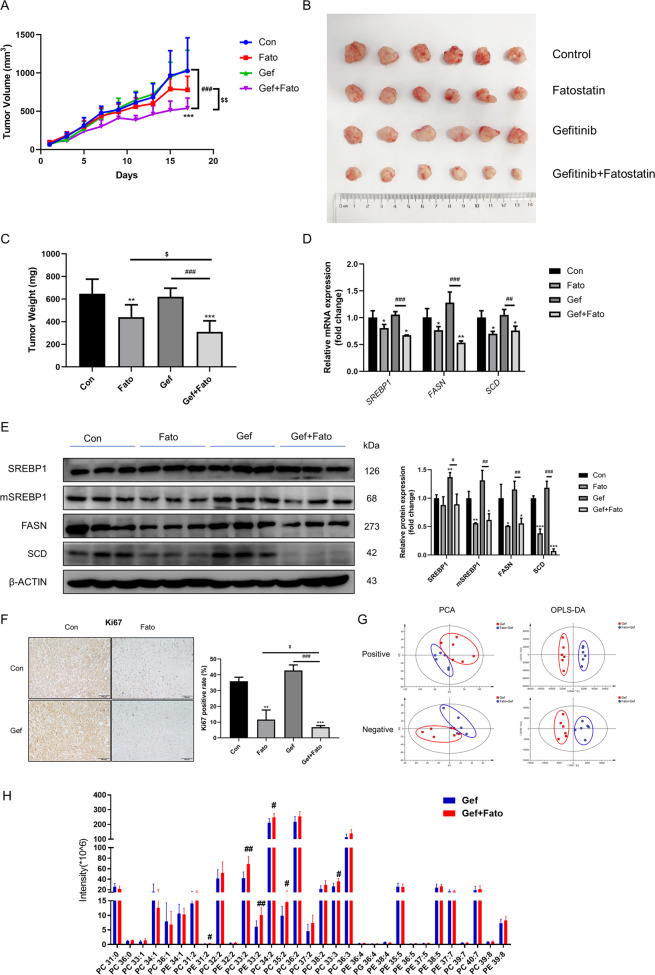
Fatostatin enhances the effect of gefitinib in PC9/GR xenograft model. Tumor-bearing BALB/c nude mice were assigned randomly into four groups (*n* = 6 per group) and administered vehicle, Gef (25 mg/kg), Fato (20 mg/kg) or a combination of Gef and Fato for 17 d (daily by oral gavage). **A** Tumor volume of PC9/GR xenografts in each treatment group. **B** Tumor from nude mice in each treatment group. **C** Tumor weight (mg) in four groups. **D** The mRNA levels of *SREBP1, FASN*, and *SCD* in tumor tissues were determined by RT-qPCR (*n* = 4). **E** The protein expression of SREBP1, mSREBP1, FASN, and SCD in tumor tissues were analyzed by western blot and the corresponding results were quantified by the density of immunoblotting (*n* = 3). **F** Immunohistochemical analysis of Ki67 in tumors and quantification were performed by Image J (*n* = 3). Scale bars indicate 100 μm. **G** Lipidomic analysis on Gef and Gef+Fato group in positive and negative ion modes, respectively. PCA and OPLS-DA score plots of lipidomic profiles obtained from LC-MS/MS. **H** Comparison of the changed lipids between Gef and Gef + Fato group. Data are expressed as the mean ± SD. *P < 0.05, **P < 0.01, ***P < 0.001 compared with control. ^*#*^*P* < 0.05, ^*##*^*P* < 0.01, ^*###*^*P* < 0.001 compared with gefitinib.

To further confirm the therapeutic benefit of fatostatin combined with gefitinib in gefitinib-resistant lung cancer, we used the xenograft models generated by grafting PC9/GR cells in nude mice. The results showed that combination of fatostatin with gefitinib could significantly suppress xenograft tumor growth, while gefitinib alone failed to inhibit tumor growth effectively during 17 d of continuous administration. The average tumor weight of gefitinib alone treatment group was 619.9 ± 76.7 mg, which was not statistically different from that of the control group, while administration of fatostatin combined with gefitinib reduced the average tumor weight to 310.2 ± 97.7 mg. Fatostatin alone also reduced tumor weight, however, the effect was more significant in the combined group (Fig. 5A–C). Immunohistochemical (IHC) staining re-confirmed that fatostatin could augments the antitumor effect of gefitinib in vivo (Fig. 5F).

Compared with the control group, the expressions of SREBP1 and downstream FASN and SCD levels did not show significant change after gefitinib was given to mice xenografted with PC9/GR cells. However, fatostatin downregulated the expression of SREBP1 and downstream target genes, whether or not combined with gefitinib (Fig. 5D, E; Fato: 11.9, 44.2, 48.7, and 61.9%; Gef + Fato:10.8, 39.4, 44.3, and 92.8%). Phospholipidomic analysis of the various treatment revealed a correlation between the changes in the poly-unsaturation of phospholipids and anti-tumor growth response (Fig. 5G, H). These data all demonstrated that inhibition of SREBP1 could strengthen the effect of gefitinib on gefitinib-resistant NSCLC cells.

## Discussion

For NSCLC patients with EGFR mutation, gefitinib is the first-line treatment recommended by NCCN, with significant therapeutic effect [23]. However, with prolonged time of treatment, most patients will develop acquired drug resistance [24]. Therefore, the biggest challenge of targeted therapy in clinical practice is to overcome the problem of drug resistance. One of the effective ways to overcome drug resistance is to identify and exploit of vulnerabilities, which are activated by and act downstream of such oncogenic pathways. Previous studies have shown that the de novo synthesis of endogenous lipids is very active in many drug-resistant tumor cells [12, 25–27]. Here, we showed that SRBEP-1 is a key feature of acquired resistance to gefitinib in EGFR mutant lung cancer and that inhibition of SREBP-1 can overcome gefitinib-acquired resistance. This study explored the mechanism of gefitinib-acquired resistance in lung cancer from the perspective of de novo lipogenesis, so as to provide a new direction to overcome gefitinib resistance.

In this study, we firstly compared the lipid groups of gefitinib-sensitive cell PC-9 and gefitinib-resistant cell PC9/GR, and found that there were significant differences in lipid groups between them. Further identification of the differential lipids resulted in 29 differential lipids, including 9 PCs, 8 PEs, 4 PGs, 4 FFAs, 2 SMs, and 2 Cers. As SREBP1 is an important transcription factor in the de novo synthesis of lipids [28], then we investigated the expression of SREBP1, FASN, and SCD, and found that they were highly expressed in drug-resistant cells, suggesting that the drug resistance of PC9/GR cells may be related to lipid metabolism. Next, we analyzed the lipid group of sensitive cells and drug-resistant cells after the treatment of gefitinib, and found that the lipid group changed significantly in these cells. The lipid levels of PC9 cells, such as Cer, SM, PG, PE, PI, PS, and FFA, were significantly changed with gefitinib treatment. PC and PE are the two types of phospholipids with the highest content in cells and are the most important structural lipids in biofilms [29]. The change in stability of phospholipid itself is the main cause of impaired cell function. Changes in the composition of the biofilm will reduce the membrane fluidity and increase the membrane permeability, thus damaging the structure and function [30]. This study for the first time found that the content of phospholipids such as PC and PE in gefitinib-sensitive PC9 cells were significantly decreased with gefitinib treatment, while the corresponding content of phospholipids in gefitinib-resistant PC9/GR cells did not decrease significantly or showed a slight increase trend. It suggested that gefitinib may inhibit the proliferation of PC9 cells by changing the composition of membrane phospholipids, but had little influence on PC9/GR cells. In gefitinib-sensitive EGFR mutant cells, the content of fatty acids decreased after gefitinib treatment, indicating that lipid synthesis was blocked. However, in gefitinib-resistant EGFR mutant cells, lipid synthesis sustained, which was similar to the situation in BRAF targeted drug-resistant cells [12].

Fatty acids are divided into saturated fatty acids, monounsaturated fatty acids, and polyunsaturated fatty acids according to the presence and number of double bonds on the R chain. In normal cells, fatty acids are generally absorbed by exogenous sources, while tumor cells are generally obtained by de novo synthesis of lipid [31]. In this study, we found that gefitinib could change the proportion of saturated phospholipids and unsaturated phospholipids in gefitinib-sensitive and drug-resistant cells. It has been reported that tumor cells can increase membrane lipid saturation through de novo lipogenesis, thus reducing the sensitivity of tumor cells to oxidative stress and common chemotherapeutic drugs [17]. These results suggest that the sustained de novo lipogenesis in gefitinib-resistant cells may affect the effect of gefitinib on its membrane phospholipids, making it less susceptible to oxidative stress and insensitive to gefitinib.

Previous studies have shown that SREBP1 and its downstream targets are highly expressed in many cancers [32–36]. Activation of SREBP-1 to synthesize lipids is thought to be required to maintain rapid proliferation of tumor cells [37]. It has been reported that lipogenesis also contributes to the resistance to cell death by altering membrane lipid composition and sensitive to lipid peroxidation [12, 38–40]. In gefitinib-sensitive PC9 cells, gefitinib caused a decrease in lipogenesis and attenuated the expression of SREBP1, mSREBP1, FASN, and SCD. However, this was not observed in gefitinib-resistant PC9/GR cells, which showed sustained levels of lipogenesis in the presence of gefitinib [41]. Together with our observation that inhibition of SREBP1 in gefitinib-resistant PC9/GR cells decreases cell growth and makes them sensitize to gefitinib in vitro and in vivo. These findings indicate that SREBP1-mediated lipogenesis is a central pathway acting downstream of mutant EGFR. SREBPs family is encoded by srebf1 and srebf2 genes [42]. Whether SREBP2 plays a role in the development of drug resistance in lung cancer cells remains to be further explored.

Our data reveals the key role of SREBP1 mediated lipogenesis in gefitinib-acquired resistance, supporting the increased interest in lipogenesis inhibition as a novel anti-neoplastic strategy. These findings imply that the combination use of SREBP1 inhibitors and gefitinib might be a new therapeutic strategy for clinical treatment of advanced lung cancer.

## Materials and methods

### Cell lines and reagents

H1975 (Cellcook Biotech, Guangzhou, China), PC9 and PC9/GR cells (Guangdong Lung Cancer Institute, Guangdong General Hospital & Guangdong Academy of Medical Sciences, Guangzhou, China) were cultured in RMPI 1640 Medium (Corning, NY, USA) containing 10% FBS (Hyclone, Utah, USA) and 1% antibiotic-antimycotic solution (Gibco, NY, USA). Resistance of PC9/GR cells to gefitinib was experimentally confirmed (Fig. 1A). Cell lines were incubated at 37 °C in a humidified atmosphere with 95% air and 5% CO_2_. BCA protein quantification kit (Thermo Fisher Scientific, MA, USA); Anti-SREBP1 (WB: 1:1000), Anti-SCD (WB: 1:1000), Anti-Ki67 (IHC: 1:50) (BBI Life Sciences, Shanghai, China); Anti-FASN (WB: 1:1000), Anti-β-Actin (WB: 1:1000), Anti-EGFR (WB: 1:1000), Anti-pEGFR (WB: 1:1000), Anti-Akt (WB: 1:1000), Anti-pAkt (WB: 1:1000) (CST, MA, USA).

### Proliferation assay

Cells were seeded in 96-well plates at a density of 5 × 10^3^ cells per well. After 24 h, the cells or siRNA-transfected cells were treated with various concentrations of compounds and incubate for 72 h. Cell viability was determined using Cell Counting Kit-8 (Dojindo, Japan) according to the manufacturer’s instructions.

### Western blot analysis

Proteins were resolved by SDS-polyacrylamide gel electrophoresis, transferred to a PVDF membrane and detected using the appropriate primary and secondary antibodies before visualization with a Chemiluminescent HRP Substrate Kit (Millipore). Visualization was performed with Image Quant LAS-4000 (Fujifilm, Tokyo, Japan) using image Multi-Gauge Software (Fujifilm). Quantification was performed by Image J.

### Measurements of cellular fatty acid, cholesterol, and triglyceride levels

The levels of cellular fatty acids (FFA), cholesterol (T-CHO), and triglyceride (TG) were quantified by the Free Fatty Acid Quantification Kit, Cholesterol Quantification Kit, and Triglyceride Colorimetric Assay Kit (Nanjing Jiancheng Bioengineering Institute, Jiangsu, China) according to the manufacturer’s recommended protocol.

### Quantitative real-time RT-PCR

Total RNA was extracted using Trizol reagent (TaKaRa Biotech, Kyoto, Japan) following the manufacturer’s protocol. Complementary DNA (cDNA) was synthesized using PrimeScriptTM RT Reagent Kit with gDNA eraser (TaKaRa Biotech) according to the manufacturer’s instructions. Sequences of primers are as follows: *SREBP1*: forward primer 5′-ACAGTGACTTCCCTGGCCTAT-3′; reverse primer 5′-GCATGGACGGGTACATCTTCAA-3′. *FASN*: forward primer 5′-AAGGACCTGT-CTAGGTTTGATGC-3′; reverse primer 5′-TGGCTTCATAGGTGACTTCCA-3′. *SCD*: forward primer 5′-TCTAGCTCCTATACCACCACCA-3′; reverse primer 5′-TCGTCTCCAACTTATCTCCTCC-3′ (BBI Life Sciences, Shanghai, China). SYBR Premix Ex-Taq ™ II Kit (TaKaRa Biotech) in a 7500 real-time PCR System (Applied Biosystems, California, USA). Relative mRNA expression was calculated by the delta delta Ct method.

### Analysis of intact phospholipid species by ESI-MS/MS

Cells were rinsed twice with 1 mL PBS, and 1 mL methanol were added into the plates. The cells were detached using a cell scraper and transferred into 15 mL tubes. Cells were mixed with 2.75 mL CHCl_3_/H_2_O (8:3, v/v). The mixture was vigorously vortexed for 3 min and centrifugated at 4000 × *g* for 5 min at 4 °C. The lower organic phase was collected respectively, and transferred into a clean tube, dried under nitrogen flow at room temperature, and stored at −80 °C until analysis. The residuals were resuspended using 300 μL acetonitrile/isopropanol (1:1, v/v) for further UHPLC-ESI-MS analysis. Phospholipids were analyzed by electrospray ionization tandem mass spectrometry (ESI-MS/MS) on AB SCIEX Triple TOFTM 5600+ (Foster City, CA), a hybrid triple quadrupole time-of-flight mass spectrometer, which is equipped with a Duo Spray Ion Source. The data were acquired using Analyst^®^ TF 1.7 software (AB Sciex, Foster City, CA).

The software SIMCA 14.1 (Umetrics AB, Sweden) was used for multivariate analysis of the original peak table data. After Parato transformation, firstly, Principal Component Analysis (PCA) was used to construct PCA (reflecting the discrete trend between groups) model. Orthogonal partial least-squares Discriminant Analysis (OPLS-DA) was used for supervised data Analysis. Variable Importance on Projection (VIP) value and confidence interval of VIP value in OPLS-DA model were selected to obtain differential metabolites (VIP > 1.5).

### Animal experiment

Male athymic BALB/c nude mice (3–4 weeks old) were purchased from Guangdong Province Medical Animal Center and fed and monitored under specific pathogen-free conditions. All animal protocols were approved by the Animal Research Committee at Sun Yat-sen University, and all treatments were administered in compliance with the National Institute of Health and Nutrition Guidelines for the Care and Use of Laboratory Animals. PC9/GR human lung cancer cells (3 × 10^7^ in 1 ml) were injected subcutaneously into the right flank of all mice and when tumor size reached 100 mm^3^, mice were randomly grouping and they were blindly administered daily by oral gavage. Mice were divided into four groups (six mice in each): (1) vehicle control; (2) 20 mg/kg fatostatin; (3) 25 mg/kg gefitinib; and (4) 25 mg/kg gefitinib and 20 mg/kg fatostatin. Fatostatin or vehicle (30% PEG (Sigma) in saline) was administered an hour after gefitinib or vehicle (1% Tween 80 (Amersham) in saline). Body weights and tumor measurements were determined every two days, and tumor volume was calculated using the formula V = (length × width^2^)/2. Animals were sacrificed and tumors were collected at the experimental endpoint, and tumors were excised for western blot and immunohistochemistry. According to the general number of experimental animals, there were six mice in each group.

### Immunohistochemical (IHC) staining

Animal tumor tissues were embedded in paraffin and sectioned at a thickness of 5 μm. Tumors slides were deparaffinized and hydrated using a graded series of xylene and ethanol solutions and washed with PBS. Antigen was retrieved in citrate buffer, and sections were immersed in 3% H_2_O_2_ to remove endogenous peroxidase activity. Then slides were incubated with primary antibodies of Ki-67 (1:50) overnight. Horseradish-peroxidase-labeled anti-rabbit IgG (CST) was subjected to DAB staining (Vector Laboratories, Burlingame, CA). After developing, all sections were observed by microscope (40×) and analyzed using the Image Pro Plus software (v.6.0) program.

### Statistical analysis

All results were expressed as means ± SD of at least three independent experiments. Statistical analyses were performed by GraphPad Prism 8 and all data are analyzed by two-tailed student’s *t*-test. Values of *P* < 0.05 were considered as being statistically significant.

## Data Availability

The datasets used and/or analyzed during the current study are available from the corresponding author on reasonable request.
